# A multi-omics approach based on ^1^H-NMR metabonomics combined with target protein analysis to reveal the mechanism of RIAISs on cervical carcinoma patients

**DOI:** 10.18632/aging.204305

**Published:** 2022-09-27

**Authors:** Chai Yanlan, Aibibai Jielili, Juan Wang, Haiying Tang, Zi Liu, Ping Wang

**Affiliations:** 1 Tianjin Medcical University Cancer Institute and Cancer Hospital, National Clinical Research Center for Cancer, Key Laboratory of Cancer Prevention and Therapy, Tianjin's Clinical Research Center for Cancer, Tianjin 300060, P.R. China; 2 Department of Radiotherapy Oncology, The First Affiliated Hospital of Xi'an Jiao Tong University, Xi'an 710061, Shaanxi, P.R. China; 3 The Gao Qing People's Hospital of Zibo, Zibo 255000, Shandong, P.R. China

**Keywords:** cervical carcinoma, RIAISs, ^1^H-NMR metabonomic, multi-omics analysis

## Abstract

Cervical carcinoma (CC) is the fourth most common cancer in females and radiotherapy is always as the definitive therapy for cervical cancer patients who are not suitable for surgery. Radiation-induced acute intestinal symptoms (RIAISs) occur in 50-80% of cervical cancer patients. Some research shows that RIAISs may relate to inflammatory reaction by radiotherapy but the action mechanism is also not clearly and the details of the molecular mechanism are still urgently needed. In this paper, basing on ^1^H-NMR metabonomic and bioinformatics analysis, an integrated multi-omics analysis including metabonomics and bioinformatics was performed. We propose a hypothesis about pathogenic mechanism on RIAISs and proofed it through western-blot. Our results indicated significant dysregulation of metabolic pathways in RIAIS patients. Most importantly, we found that RIAISs were associated p53 and PI3K-AKT pathway.

## INTRODUCTION

During the therapeutic process of patients with cervical cancer and patients who are adverse surgical candidates, radiotherapy is always the ultimate treatment [1]. With advances in radiotherapy and related treatments, significant progress has been made in high- accuracy radial delivery and radiation therapy dose distribution. However, complication and the influence to normal tissue remains an issue [2].

The intestine is particularly sensitive to ionizing radiation and causes side effects such as vomiting, anorexia, weight loss, diarrhea, dehydration, and infection [3]. Patients receiving radiation to the cervix, pelvis, abdomen, and colorectal system often experience short-term enteritis, but most experience chronic bowel problems [4]. When exposed to ionizing radiation, it causes mucosal inflammation and other side effects in pelvic radiotherapy. Radiation induced intestinal damage (RIID) will affect the treatment schedule and decrease patients’ quality of life [5].

Cervical carcinoma (CC) is the fourth most common cancer in females, especially in developing countries [6]. However, radiotherapy can almost cause radiation-induced acute intestinal symptoms (RIAISs) in patients with cervical cancer. Some researches show that RIAISs may related to inflammatory reaction by radiotherapy but the action mechanism is also not clearly and the details of the molecular mechanism are still urgently needed [7]. Currently, the treatment of RIIAS is mainly focused on supportive and complementary treatment, and the current treatment goal is still only to reduce the severity of symptoms and exacerbations [8].

Metabolomics is becoming an important component of systems biology [9]. Currently, metabolomics has been successfully applied to disease diagnosis, biomarker screening, and biological pathway characterization [10]. Except the mass spectrometry, the technological advances in nuclear magnetic resonance (NMR) spectroscopy have further improved the sensitivity and spectral resolution of metabolomics studies. NMR has irreplaceable advantages such as allowing non-destructive methods in untargeted metabolic analysis with little or no sample preparation [11, 12]. Now NMR spectroscopy is a powerful tool that is widely applied in metabolite identification and quantification [13].

In this study, feces, serum, and urine of CC patients were used as samples. An ^1^H NMR based metabonomic analyses was employed to detect the change of metabolins before and after radiotherapy between different groups. The data integrated with bioinformatics analysis results were used to identify potential targets or pathways and novel therapeutic regimens. As a result, we built a profile of RIAISs which based on metabolins and bioinformatics. We found that radiotherapy mainly affected the PI3K-AKT and P53 pathway. The activation of these pathways will lead to an inflammatory reaction and these results could reveal the nosogenesis of RIAISs. The result will aid in our understanding of the pathobiology of the disease.

## MATERIALS AND METHODS

### Reagents and materials

Chemiluminescent HRP substrates were purchased from Millipore Corporation (MA, USA). Primary antibodies against AKT (33748), p-AKT (11054), NFKB (48498), p-NFKB (11166), GAPDH (40493), goat anti-rabbit IgG secondary antibody (L3012) were purchased from Signalway Antibody (MA, USA).

### Patients’ treatment, sample collection and storage

Detail information are as the same as our previous study [14]. A summary of the patients’ characteristics is displayed in Table 1.

**Table 1:** Clinical and demographic information on all 66 cervical cancer patients, 11 of whom developed RIAISs.

**Characteristics**	**Participants**	**RIAISs**	**Controls**
No.of women	66	11	11
Age (years)			
Median/mean	53/52.1	54/51.9	55/54.2
Range	30-70	31-67	37-65
FIGO stage			
I	10	2	2
II	37	6	7
III	13	2	2
IV	6	1	0
Histological type			
Squamous carcinoma	64	11	11
Adenocarcinoma	2	0	0
Therapy			
Radical hysterectomy and adjuvant radiotherapy	34	7	7
Radical radiotherapy	32	4	4

On treatment of patients, our previously published treatment protocol was followed for this study [14]. The details of patients’ treatment, sample collection and storage are shown in the supporting information.

Samples were grouped as follows:

Group I-samples of RIAIS group before radiotherapy.
Group II-samples of the control group before radiotherapy.
Group III-samples of RIAIS group after radiotherapy.
Group IV-samples of the control group after radiotherapy.

### Specimen preparation for ^1^H NMR analysis

**Figure 1:**
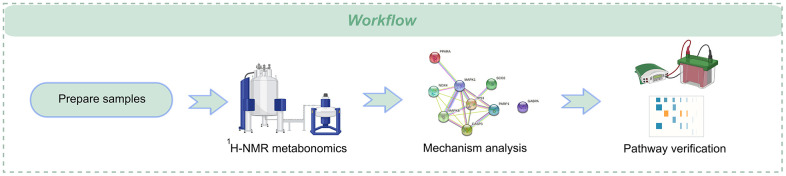
The workflow of the whole study.

This section was according to our previous work [14]. The details of specimen preparation for ^1^H NMR analysis are shown in the supporting information. The workflow of the whole study is shown in Figure 1.

### ^1^H NMR analysis

This section was according to our previous work [14]. Spectra primarily comprising signals from metabolites and small molecules to be obtained [15]. The details of ^1^H NMR analysis are shown in the supporting information.

### Multivariate statistics

This section was according to our previous work [14]. The details of multivariate statistics are shown in the supporting information.

### Metabolomic identification and bioinformatics analyses

To identify the differential metabolites and pathway ascription, a corresponding metabolomic database (http://hmdb.ca) and Compound Discoverer 3.0 software was used as the search database. Next, we used the DAVID tool (https://david.ncifcrf.gov/) to analyze protein function and pathway enrichment. To identify the key targets, we used Venny 2.1.0 to intersect two parts, including all proteins from differential pathways by metabonomic analysis, proteins associated with RIAISs by GeneCards database. Then, the key target was uploaded to the STRING database (https://www.string-db.org/cgi/input.pl), and the minimum required interaction score was set to 0.4 to obtain a protein–protein interaction (PPI) network of key targets and proteins related to the key targets. Finally, the constructed networks were merged, and the duplicated edges were removed and visualized in Cytoscape3.2.1.

### Western blot

Total pulmonary protein was extracted from 50 mg of frozen urine sample. Then the total proteins either from cells or tissues by RIPA lysis buffer were applied for the western blot analysis, which processed in accordance with our previous methods [16].

### Statistical analysis

The results are shown as the mean ± standard deviation (SD). For single comparisons, significant differences between the means were determined by one way ANOVA test. A p-value less than 0.05 was considered to indicate significant differences. All data were processed using GraphPad Prism 5.01 software.

## RESULTS

### ^1^H NMR spectrum and PCA analysis

**Figure 2:**
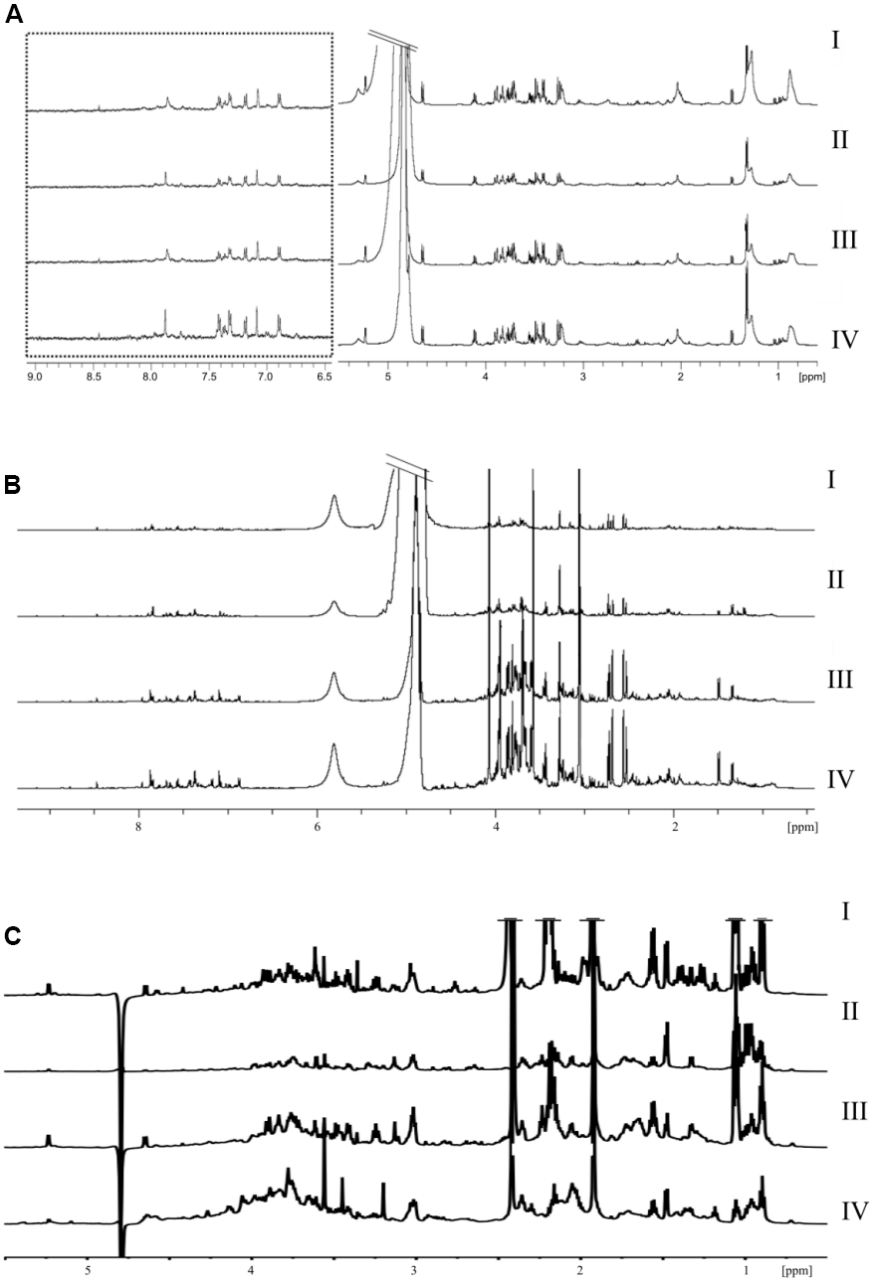
**NMR chromatograms different samples and groups.** (**A**) plasma (**B**) urine (**C**) feces.

**Figure 3:**
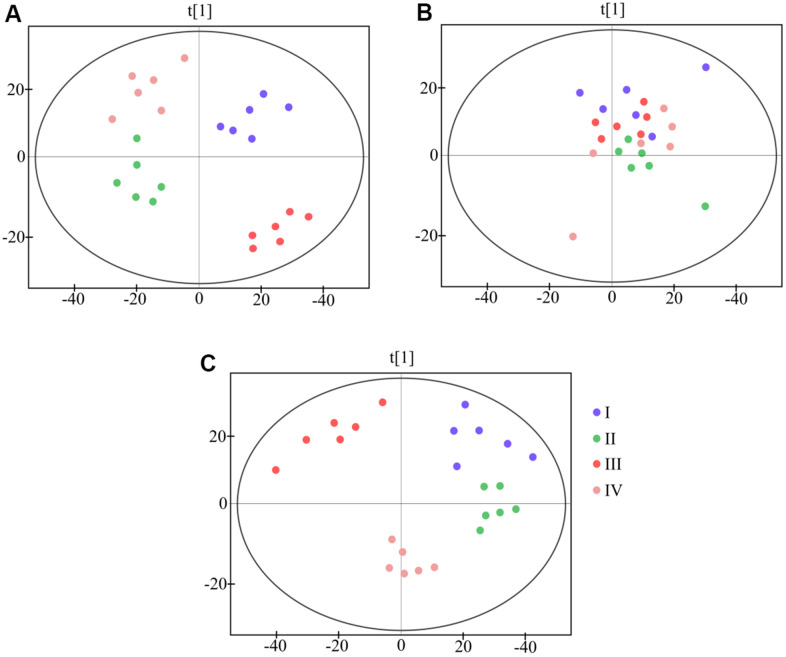
**PCA scores plot based on ^1^H NMR spectra of different sample from four groups.** (**A**) plasma (**B**) urine (**C**) feces.

Import ^1^H NMR results into MestReNova software for phase correction and baseline adjust. Figure 2 shows the signals from different samples and groups. To establish a global overview for discrimination between different groups, we did a PCA analysis of all NMR spectral data between different groups (Figure 3), all groups have separation trends expect the urine sample.

### Metabonomic analyses revealed the key pathways of RIAISs caused by radiotherapy

**Figure 4:**
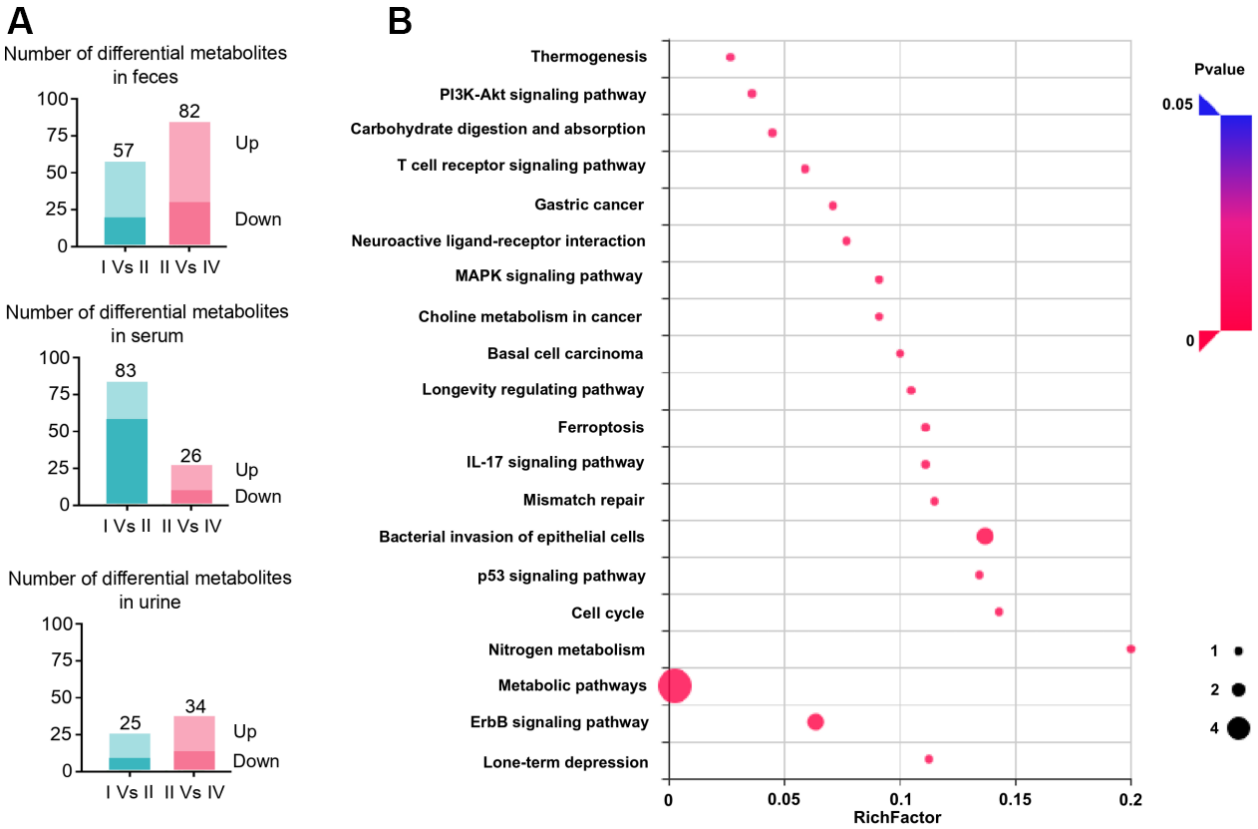
**The integral analysis of quantitation proteomics, metabonomics and COPD associated proteins.** (**A**) ^1^H NMR-based metabolomic analysis of faces, serum, and urine sample. Quantitation of metabolites with the condition of Fold-change ≥1.2 or ≤0.83 and p-value ≤0.05. (**B**) KEGG pathway enrichment analysis of differential metabolites.

To explore the underlying molecular mechanisms, an ^1^H NMR-based metabolomic strategy was first used to identify differential metabolites in CC patients after radiotherapy. As a result, 733 metabolites were identified with a false discovery rate (FDR) ≤ 0.01. After further data analysis by HMDB database, a total of 524 metabolites were found to be significantly different among different groups. With the criteria of p-value < 0.05, only 151 metabolites were found. These proteins were annotated in 20 different pathways in the KEGG database. Among these findings, the PI3K-Akt, MAPK, Thermogenesis, Choline metabolism in cancer, IL-7 metabolic pathways, p53, and other pathways were involved the main biological processes of RIAISs (Figure 4A, 4B).

**Figure 5:**
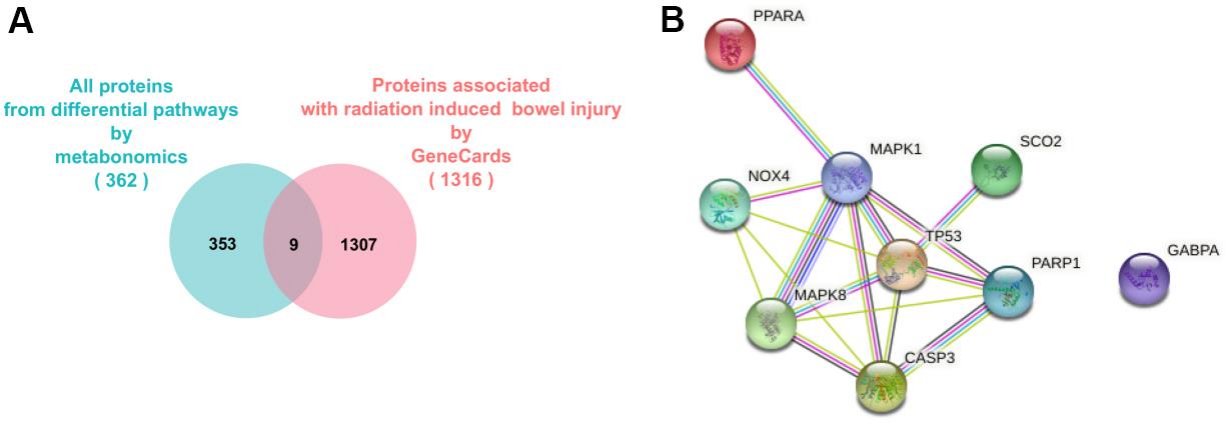
**Network pharmacological analysis of RIAISs caused by radiotherapy.** (**A**) Strategy of find out key target. (**B**) Connection network between the key nodes calculated by STRING.

To clarify the key action nodes of RIAISs caused by radiotherapy, all associated proteins involved in above differential pathways, which were identified by metabonomics (362) and RIAISs disease-associated proteins (1316) obtained from GeneCards, were used in the integrated analysis. As shown in the Venn diagram in Figure 5A, only 9 intersection node proteins were identified: NFKB1, RAF1, TP53, AKT1, SCO2, MDM2, TLR3, PIK3CA, MAPK3 and Figure 5B shows the association of these proteins.

### Network analysis of RIAISs caused by radiotherapy

To further predict the molecular mechanism of RIAISs caused by radiotherapy, a PPI network was built. As Figure 3A shows, there were a total of 58 proteins that interacted with the key proteins above and these proteins were regulated by the 9 focused proteins in the protein-protein interaction network. The 9 focused proteins could be divided into 19 signaling pathways like p53 signaling pathway, PI3K-Akt signaling pathway, Apoptosis - multiple species, Colorectal cancer and so on. Table 2 shows the fit score of rank of different pathways which exported by DAVID database. Significantly, the PI3K-Akt signaling pathway and p53 signaling pathway ranked the first and second. It prompts us that the possible molecular mechanism of RIAISs caused by radiotherapy.

**Table 2:** KEGG pathway enrichment analysis of key nodes.

**No.**	**Pathway**	**Strength**
1	p53 signaling pathway	2.15
2	PI3K-Akt signaling pathway	2.13
3	Colorectal cancer	2.06
4	Apoptosis - multiple species	1.91
5	Central carbon metabolism in cancer	1.87
6	IL-17 signaling pathway	1.82
7	MicroRNAs in cancer	1.82
8	Ras signaling pathway	1.81
9	cAMP signaling pathway	1.8
10	Th1 and Th2 cell differentiation	1.8
11	Choline metabolism in cancer	1.78
12	Sphingolipid signaling pathway	1.75
13	GnRH signaling pathway	1.72
14	Toll-like receptor signaling pathway	1.69
15	Natural killer cell mediated cytotoxicity	1.67
16	Wnt signaling pathway	1.65
17	Cellular senescence	1.61
18	Necroptosis	1.58
19	Pathways in cancer	1.58

**Figure 6:**
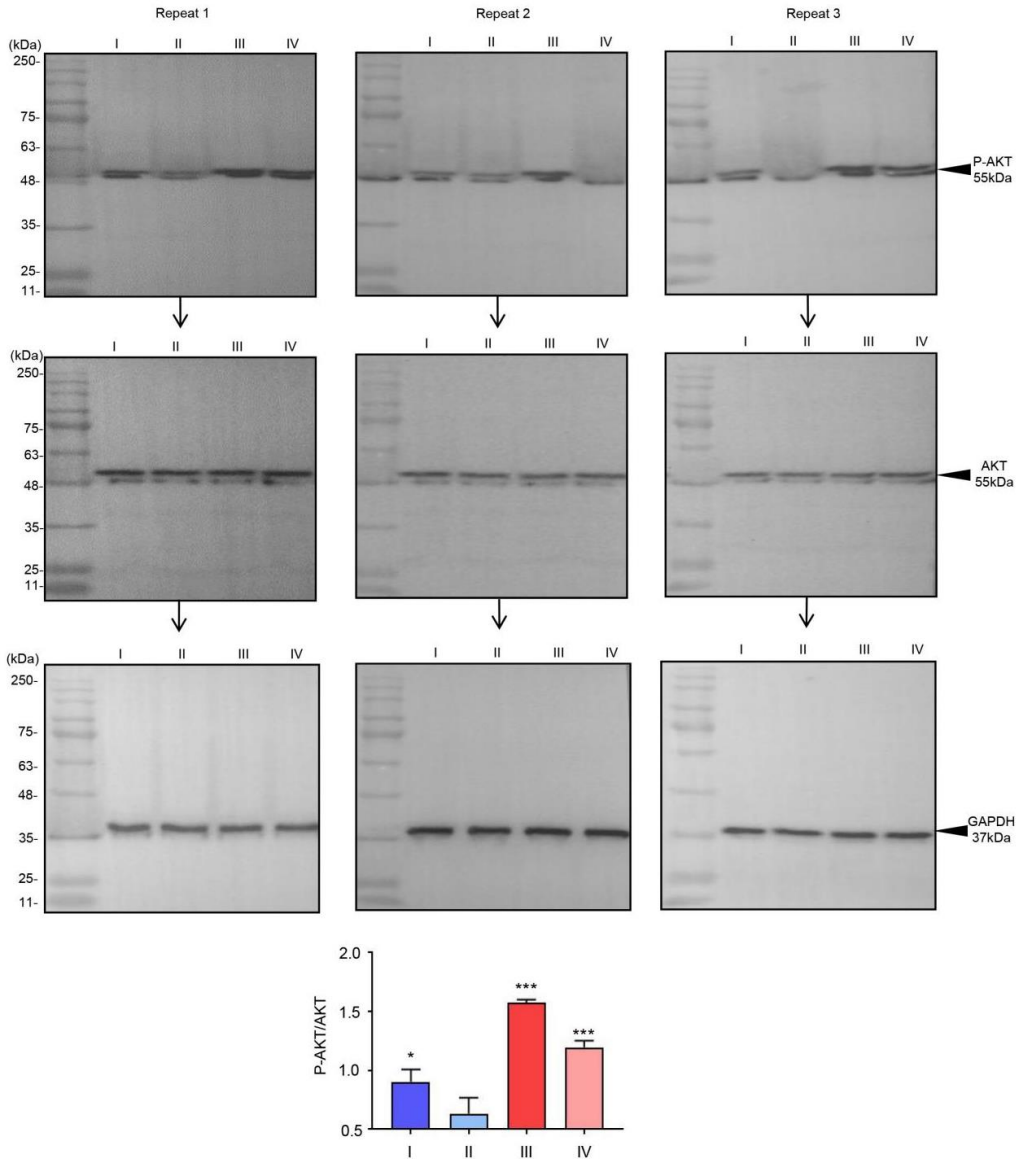
**The phosphorylation of AKT in urine sample.** Phosphorylation of AKT in urine sample regulated by radiotherapy. The relative intensity of P-AKT was compared with AKT.^*^p< 0.05, and ^***^p< 0.001 vs. II, (n = 3).

**Figure 7:**
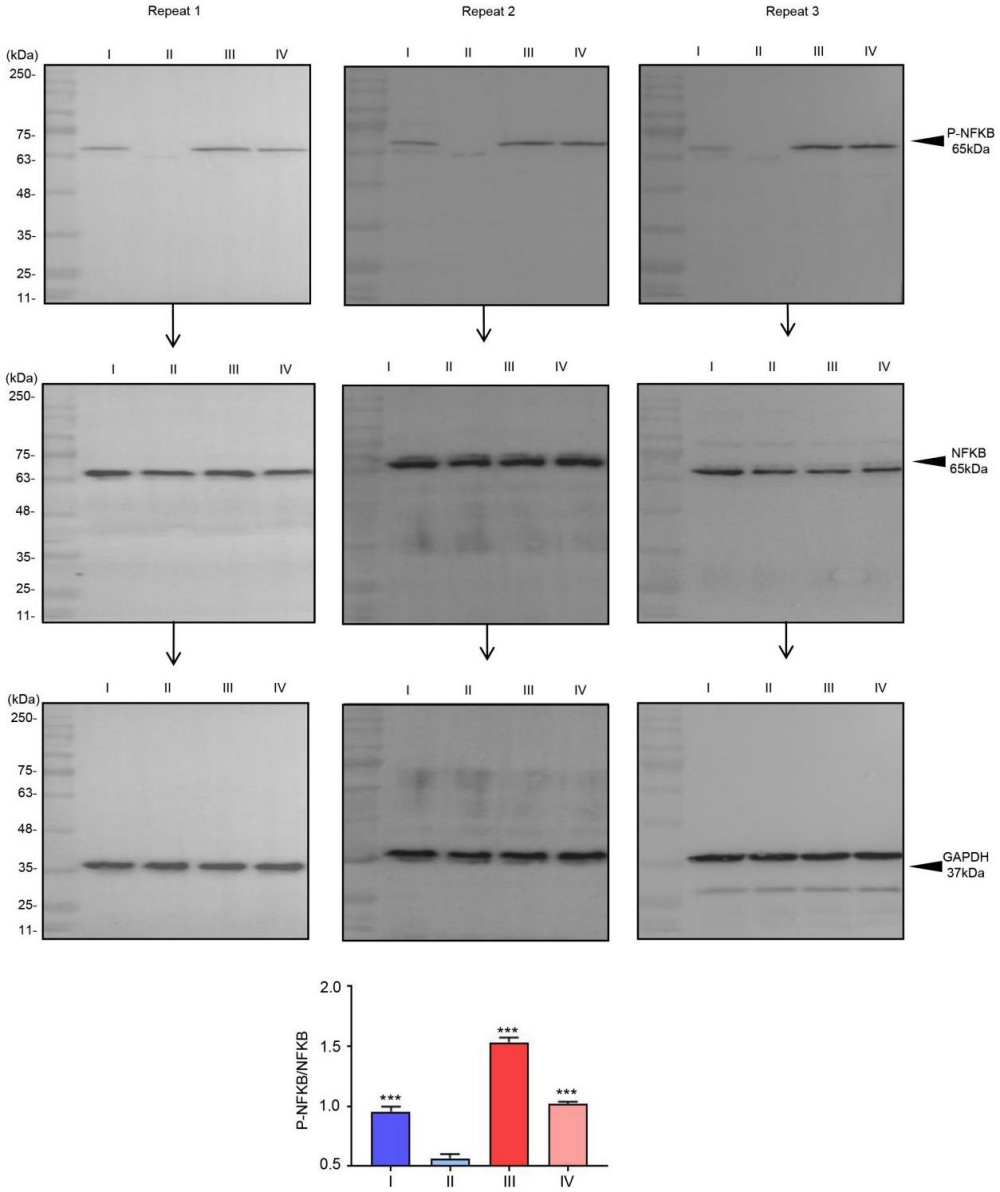
**The phosphorylation of NFKB in urine sample.** Phosphorylation of NFKB in urine sample regulated by radiotherapy. The relative intensity of P-NFKB was compared with NFKB. ^***^p< 0.001 vs. II, (n = 3).

According the results about the network analysis above, we choose the downstream proteins in PI3K-Akt signaling pathway and p53 signaling pathway and tried to test and verify the phosphorylation levels of AKT and NFKB. The changes in the phosphorylation levels of AKT and NFKB in the urine samples are shown in Figures 6, 7. Western blot analysis revealed that, after radiotherapy the phosphorylation levels of AKT and NFKB were markedly increased (Tables 3, 4), it indicated that radiotherapy activate the PI3K-Akt and p53 signaling pathway.

**Table 3:** Raw data of western blotting with P-AKT in clinical tissue samples.

**Group**		**I**	**II**	**III**	**IV**
P-AKT	repeat1	1846	1362	2899	2314
	repeat2	1460	899	2825	2126
	repeat3	1669	1273	2963	2177
AKT	repeat1	3065	3076	3044	3048
	repeat2	3059	3100	3028	3077
	repeat3	3008	3088	3074	3060
GAPDH	repeat1	4290	4098	4001	4123
	repeat2	4137	4199	4241	4211
	repeat3	4087	4171	4164	4425
P-AKT/AKT	repeat1	1.00	0.74	1.58	1.26
	repeat2	0.79	0.48	1.55	1.15
	repeat3	0.92	0.68	1.60	1.18

**Table 4:** Raw data of western blotting with P-NFKB in clinical tissue samples.

**Group**		**I**	**II**	**III**	**IV**
P-NFKB	repeat1	1996	1063	2962	2027
	repeat2	1887	954	3088	2074
	repeat3	1811	960	3082	2011
NFKB	repeat1	3065	3076	3044	3048
	repeat2	3059	3100	3028	3077
	repeat3	3008	3088	3074	3060
GAPDH	repeat1	3969	4111	3949	4077
	repeat2	4109	4291	3902	4139
	repeat3	3948	4081	4012	3912
P-NFKB/NFKB	repeat1	1.00	0.53	1.49	1.02
	repeat2	0.95	0.4	1.57	1.04
	repeat3	0.92	0.48	1.54	1.01

## DISCUSSION

Radiotherapy is commonly used as the ultimate treatment for patients with locally advanced cervical cancer or poor surgical candidates [17]. For cervical cancer patients, the intestine is a major dose-limiting organ during pelvic radiotherapy because the intestine is sensitive to ionizing radiation [18]. RIAISs occurs in 50–75% of cervical cancer patients after radiotherapy, it will lead dose reductions, treatment interruptions, increased medical costs, and reduced quality of life, with symptoms including abdominal pain, diarrhea, and tenesmus. So, it is eagerly to find the molecular markers of RIAISs and supplement the details of molecular mechanism of RIAISs and it will help us improve the planning and monitoring of radiotherapy [19].

^1^H NMR spectroscopy is a powerful tool for chemical structural elucidation and chemical dynamic studies and analytic instrument to get information of biomarkers and help us reveal the mechanism of disease more accurately [20]. Furthermore, it will help us understand different stratification of patients and identify specific drugs that respond better. It also provides a bridge between preclinical disease models and human patient populations, where common biomarkers and patient populations between model systems provide important mechanistic connections and highlight potential therapeutic goals. This is the basis of precision drug therapy [21].

However, although the accuracy of instruments is developing rapidly, but single-omic results still have some limits, which mainly focus on the one-sidedness description of the entire life system. Bioinformatics now offered us more and more useful tools and databases, they could be a supplement of single-omic results and they will help us describe the vital process in a integrity view. In this study, a reasonable analysis strategy which based on metabolins and bioinformatics was used [22]. We found that radiotherapy mainly affected the PI3K-AKT and P53 pathway.

P53 is a tumor suppressor gene and is one of the primary cellular factors determining the nature of growth arrest and/or cell death after exposure to ionizing radiation. P53 pathway plays an important role in damage surveillance and as such has been dubbed the guardian of the genome. Upon DNA damage, P53 pathway was activate, downstream proteins (like NFKB) accumulation was increased and translocated to the nucleus where it binds to DNA, acting to regulate the transcription of a number of genes [23]. According to our results, after radiotherapy, the phosphorylation levels of NFKB were markedly increased, it indicated that radiotherapy activate the p53 signaling pathway.

PI3K-AKT signaling plays a significant role in oxidative stress and inflammation [24]. In addition, it also plays a key role in apoptosis and proliferation of tumor metastasis and invasion by regulating several pathways associated with cell growth, apoptosis, or invasion [25]. Activated AKT translocates to the cytoplasm and nucleus and activates downstream targets involved in survival, proliferation, cell cycle progression, growth, migration, and angiogenesis [26]. According to our results, after radiotherapy, the phosphorylation levels of AKT was markedly increased, it indicated that radiotherapy activate the PI3K-AKT signaling pathway.

In summary, this study demonstrated distinct differences in the spectra acquired between the feces of RIAIS patients before and after radiotherapy, and between RIAISs and controls. Basing on a ^1^H-NMR metabonomic and bioinformatics analysis, we proposed a hypothesis about pathogenic mechanism on RIAISs and proofed it through western blot. Our results indicate significant dysregulation of metabolic pathways in RIAIS patients. Most importantly, we found that RIAISs were associated p53 and PI3K-AKT pathway.
